# The contribution of evolvability to the eco‐evolutionary dynamics of competing species

**DOI:** 10.1002/ece3.10591

**Published:** 2023-10-11

**Authors:** Anuraag Bukkuri, Kenneth J. Pienta, Sarah R. Amend, Robert H. Austin, Emma U. Hammarlund, Joel S. Brown

**Affiliations:** ^1^ Cancer Biology and Evolution Program, Department of Integrated Mathematical Oncology Moffitt Cancer Center Tampa Florida USA; ^2^ The Brady Urological Institute Johns Hopkins School of Medicine Baltimore Maryland USA; ^3^ Department of Physics Princeton University Princeton New Jersey USA; ^4^ Tissue Development and Evolution Research Group, Department of Laboratory Medicine Lund University Lund Sweden

**Keywords:** adaptive dynamics, adaptive radiation, eco‐evolutionary dynamics, evolutionary rescue, evolutionary tracking, evolvability, G functions

## Abstract

Evolvability is the capacity of a population to generate heritable variation that can be acted upon by natural selection. This ability influences the adaptations and fitness of individual organisms. By viewing this capacity as a trait, evolvability is subject to natural selection and thus plays a critical role in eco‐evolutionary dynamics. Understanding this role provides insight into how species respond to changes in their environment and how species coexistence can arise and be maintained. Here, we create a G‐function model of competing species, each with a different evolvability. We analyze population and strategy (= heritable phenotype) dynamics of the two populations under clade initiation (when species are introduced into a population), evolutionary tracking (constant, small changes in the environment), adaptive radiation (availability of multiple ecological niches), and evolutionary rescue (extreme environmental disturbances). We find that when species are far from an eco‐evolutionary equilibrium, faster‐evolving species reach higher population sizes, and when species are close to an equilibrium, slower‐evolving species are more successful. Frequent, minor environmental changes promote the extinction of species with small population sizes, regardless of their evolvability. When several niches are available for a species to occupy, coexistence is possible, though slower‐evolving species perform slightly better than faster‐evolving ones due to the well‐recognized inherent cost of evolvability. Finally, disrupting the environment at intermediate frequencies can result in coexistence with cyclical population dynamics of species with different rates of evolution.

## Graphical abstract

We develop an evolutionary game theoretic model to investigate the role of evolvability in the eco‐evolutionary dynamics of species. We investigate scenarios in which either the slow‐ or fast‐evolving species go extinct and show other scenarios in which coexistence is possible.

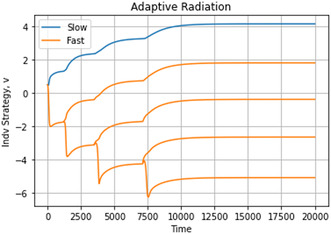

## 1 INTRODUCTION

In this paper, we use evolutionary game theory to model competition between a slow‐ and a fast‐evolving species. Evolvability, the ability of an organism to generate heritable variation that can be acted upon by natural selection, plays a central role in how population sizes and trait characteristics change over time. Intuitively, generating large amounts of heritable variation permits a species to rapidly evolve in response to novel environmental stressors or avoid extinction following drastic environmental changes. However, in stable environments, species that are close to their fitness peaks gain little from generating heritable variants farther from the peak. Low evolvability would permit such a species to evolve toward this peak without producing too many unfit individuals with potentially deleterious or even lethal mutations.

Theoretically, many modeling efforts have indirectly investigated the role of evolvability. Primarily, this has been done through mutation‐selection models and balancing selection models (Barton, 1999; Charlesworth & Mayer, 1995; Felsenstein, 1979; Haldane & Jayakar, 1963; Podolsky, 2001). These models tend to be relatively mechanistic and tied to the genetic details of the organism and/or problem under consideration. Furthermore, the cause of selection is typically secondary (if considered at all). Kinetic models of replication and mutation have explored RNA replication (Biebricher et al., 1983, 1984, 1985). In this approach, replication and mutation are viewed as chemical reactions that can be analyzed at the molecular level and extended to develop a theory of evolution based on biochemical kinetics (Schuster, 2011). The dependence of error thresholds that provide an upper bound for (uniform) mutation rates (Eigen, 1971) on the underlying fitness landscape in sequence space has been considered (Phillipson & Schuster, 2009; Wiehe, 1997).

Here, we follow the traditions of quantitative genetics (Falconer & Mackay, 1996) and adaptive dynamics (Dieckmann & Law, 1996; Geritz et al., 1998) by giving the faster‐evolving species greater heritable variation on which natural selection can act. While we do not explicitly consider the mechanisms for maintaining variation, a number of mechanisms exist for generating heritable variation at faster or slower rates. For example, Colegrave and Collins (2008) propose three categories of traits contributing to evolvability: traits influencing mutation rates or gene repair (e.g., increased mutation rates; Colegrave & Collins, 2008; Earl et al., 2004; Richard Moxon et al., 1994), traits that increase genetic variation through the exchange of genetic material between lineages (e.g., eukaryotic sex or horizontal gene transfer; Chen & Dubnau, 2004; Colegrave & Collins, 2008; Hawkey & Jones, 2009; Johnsborg et al., 2007; Redfield, 2001), and traits that modify genetic architecture such as the structure of gene networks (Colegrave & Collins, 2008; Hansen, 2006; Wagner, 2005; Wagner & Altenberg, 1996).

Here, we develop a simple and comprehensive model based on evolutionary game theory to examine the effect of evolvability on eco‐evolutionary dynamics. We use our model to investigate the conditions under which being less or more evolvable is favorable. Specifically, we compete for species with low and high evolvabilities against each other under four evolutionary scenarios: clade initiation, evolutionary tracking, evolutionary rescue, and adaptive radiation (see Box 1 below). For these scenarios, we simulate population (ecological) and strategy (evolutionary) dynamics. Furthermore, we visualize these dynamics on adaptive landscapes to clearly observe how the fitness of the species changes over time as a function of their evolutionary strategy (Bukkuri & Brown, 2021). This work develops a hitherto missing general, abstract model to probe how evolvability impacts eco‐evolutionary dynamics and serves as a launching point into more in‐depth investigations of the multifaceted role of evolvability in various ecological and evolutionary settings. Being able to conceptualize and model the four scenarios has taken on greater significance in light of rapid evolution of species in response to human disturbances (González‐Tokman et al., 2020; Grainger & Levine, 2022).

BOX 1 **DESCRIPTION OF EVOLUTIONARY SCENARIOS**

In this paper, we investigate the role of evolvability under the eco‐evolutionary dynamics of species in the following four scenarios:

*Clade initiation*: Species are introduced into a new environment, for example, due to migration from a mainland to an island, or as an invasive species. The environment itself is stable and unchanging, but the species may be far from their evolutionary optima (evolutionarily stable strategy, ESS) within their ecological context.

*Evolutionary tracking*: Constant changes in the environment: these changes can be stochastic (e.g., sudden and recurring algal blooms that lead to oxygen deficiency in deep waters) or deterministic (e.g., the passing of seasons which changes the local climate, or even global climate change) in nature. Species in these environments experience continual shifts in the peaks of their adaptive landscape and are thus constantly chasing moving evolutionary peaks.

*Evolutionary rescue*: Species may face severe environmental catastrophes such as deforestation or abrupt changes to their climate or physical environment, which leave them far from their evolutionary peaks. The current strategies of the species are no longer viable. Only those that can rapidly evolve a viable strategy can remain extant.

*Adaptive radiation*: The availability of multiple niches allows for species to occupy different ecological niches and avoid competition from one another, thereby leading to diversification (e.g., Darwin's finches where the diversity of food sources led to radiation of a single species into numerous species with diverse body sizes and beak morphologies) (De León et al., 2014; Foster et al., 2008).

## 2 MODEL FORMULATION

First, we construct a general, unifying model to test the contributions of evolvability to the eco‐evolutionary dynamics of two competing species, one with low evolvability and the other with high. Other than having different evolvabilities, these two species will be entirely identical. To do this, we use the G function approach (Bukkuri & Brown, 2021; Vincent & Brown, 2005). This approach provides a simple framework to model the ecological and evolutionary dynamics of interacting species in the form of a series of coupled ODEs. Critically, it does not require a particularly mechanistic formulation, allowing for broad generality and applicability.

The expected fitness or per capita growth rate of a focal individual of each species, $G\left(v,u,x\right)$, is governed by its heritable trait (strategy), *v*, the traits of the others in the population represented by a vector **u** = ($u_{1}$, $u_{2}$), where $u_{1}$ and $u_{2}$ are the trait values for the fast‐ and slow‐evolving species, respectively, and the vector $x=\left(x_{1},x_{2}\right)$ gives the population sizes of the fast‐ and slow‐evolving species, respectively. The population dynamics of species i is given by the product of the current population size and the fitness‐generating function, G:

(1) $$\frac{dx_{i}}{dt}=x_{i}G\left|{}_{v=u_{i}}.\right.$$

According to Fisher's fundamental theorem of natural selection (Basener & Sanford, 2018; Frank & Slatkin, 1992; Lessard, 1997; Li, 1967), the rate of evolution is proportional to the additive genetic variance, as produced by evolvability, multiplied by the strength of selection. Thus, mathematically, the rate of change in a species' trait value can be formalized as follows:

(2) $$\frac{du_{i}}{dt}=k_{i}\frac{dG}{dv}\left|{}_{v=u_{i}}\right.,$$

where $k_{i}$ is a measure of heritable variation (the trait's evolvability), and dG/dv is the selection gradient. Thus, the fast‐evolving species has a larger k than the slow‐evolving species: $k_{1}>k_{2}$.

To concretely investigate how evolvability impacts the fitness of competing species, we use a series of ordinary differential equations to model their population and strategy dynamics. As our modeling base, we start with a fitness‐generating function based on the Lotka–Volterra competition equations:

(3) $$G\left(v,u,x\right)=\frac{r}{K\left(v\right)}\left[K\left(v\right)-\sum_{j=1}^{2}a\left(v,u_{j}\right)x_{j}\right]-dk,$$

where carrying capacity is a function of the focal individual's strategy, v. We allow for the incorporation of competition between individuals through the $a\left(v,u_{j}\right)$ term in which the competition that species experience from each other depends on their strategies. We assume that there exists a cost (d) of evolvability, that is, there is a penalty for generating large amounts of heritable variation. We make the simplifying assumption that this penalty scales linearly with evolvability, but further investigation into different, biologically mechanistic functional forms for this cost is warranted. We use the following form for carrying capacity:

(4) $$K\left(v\right)=K_{m}\exp\left[-\frac{v^{2}}{2\sigma_{k}^{2}}\right].$$

This form assumes that when v=0, a species maximizes its carrying capacity. For values smaller or larger than v=0, carrying capacity declines according to a Gaussian distribution with a breadth of $\sigma_{k}^{2}$. We choose v=0 as the maximum point as it provides an easy reference point (any additive shift makes no difference). For the competition function, we use

(5) $$a\left(v,u_{i}\right)=\exp\left[-\frac{{\left(v-u_{i}\right)}^{2}}{2\sigma_{a}^{2}}\right].$$

This form assumes that like competes most with like for resources and space. The amount of competition individuals experience from each other declines in a Gaussian fashion as strategies diverge from each other with a breadth of $\sigma_{a}^{2}$. Thus, two individuals will compete more if their trait values are similar rather than dissimilar. The competition function has the property that when a focal individual's trait equals that of its competitor, $v=u_{i}$, then the competition coefficient is unity: $a\left(v,u_{i}\right)=1$. The parameter values used in the following simulations, unless otherwise specified, are as given in Table 1. For each evolutionary scenario, simulations were performed by solving our system of ODEs numerically using Python's odeint solver for three initial strategy conditions: $u\left(0\right)=0.5$, $u\left(0\right)=4$, and $u\left(0\right)=10$. We refer to these initial strategy conditions as the close, medium, and far cases, in relation to the strategy equilibrium of $u_{1}=u_{2}=0$.

**TABLE 1:** Parameter values used in simulations.

Parameter	Interpretation	Value
$K_{m}$	Maximum carrying capacity	100
d	Cost of evolvability	0.05
r	Intrinsic growth rate	0.25
$k_{1}$	Fast evolvability	0.5
$k_{2}$	Slow evolvability	0.2
$\sigma_{k}^{2}$	Environmental niche size	12.5
$\sigma_{a}^{2}$	Species niche width	100

Before we simulate these Darwinian dynamics and investigate the population and strategy dynamics of competing species, we will analytically derive the conditions for the population and strategy equilibria. At an eco‐evolutionary equilibrium of $u^{*}$ and $x^{*}$, the per capita growth rate of each extant species (those with positive population sizes, $x_{i}^{*}>0$) will be zero:

(6) $$G\left(v,u^{*},x^{*}\right)=0\;\text{for}\;\mathrm{all}\;{v=u}_{i}^{*},$$

and the evolutionarily stable trait value of each extant species, $u_{i}^{*}$, will reside on a peak of the adaptive landscape. Specifically, if the number of evolutionarily stable species are given by n, then the adaptive landscape will have multiple peaks and each $u_{i}^{*}$ will reside on its own unique peak separated from others by at least one valley of the adaptive landscape. This means that, from the ESS maximum principle, the following conditions must hold:

(7) $$\begin{array}{ll} \frac{dG}{dv} & =0\;\text{for}\;\mathrm{all}\;{v=u}_{i}^{*},\\ \frac{d^{2}G}{dv^{2}} & <0\;\text{for}\;\mathrm{all}\;{v=u}_{i}^{*}. \end{array}$$

This guarantees that the species will be at an ecological equilibrium and at an evolutionary equilibrium, at a local peak on its adaptive landscape.

## 3 CLADE INITIATION

When a species enters a new environment, the trait value of the species, $u_{i}$, may be most likely initially far from its eco‐evolutionary equilibrium. This would be the case, for example, when migrating from a mainland to an island. Barring such conditions that the species is so maladapted to its new environment that it cannot survive, we can expect its population size to grow: ${\left.G\left(v,u_{i},x_{i}\right)\right|}_{v=u_{i}}>0$ and its trait value to evolve: ${\left.dG/dv\right|}_{v=u_{i}}\neq 0$. If we compete with the high evolvability and low evolvability species in this scenario, we hypothesize that the fast‐evolving species will come out as the victor, assuming their initial trait values ($u_{1}$ and $u_{2}$) are far from the eco‐evolutionary equilibrium. Initially, they both reside at the same point on their respective adaptive landscapes. But the fast‐evolving species will evolve up its landscape more rapidly leaving the slow‐evolving species with a lower fitness because it is now downslope from the first. However, if they instead start close to the eco‐evolutionary equilibrium, the slow‐evolving species will outcompete the fast evolving one since the latter, due to its high evolvability and thus variance in progeny trait values, will produce many progenies off the peak, leading to a lower fitness.

**FIGURE 1:**
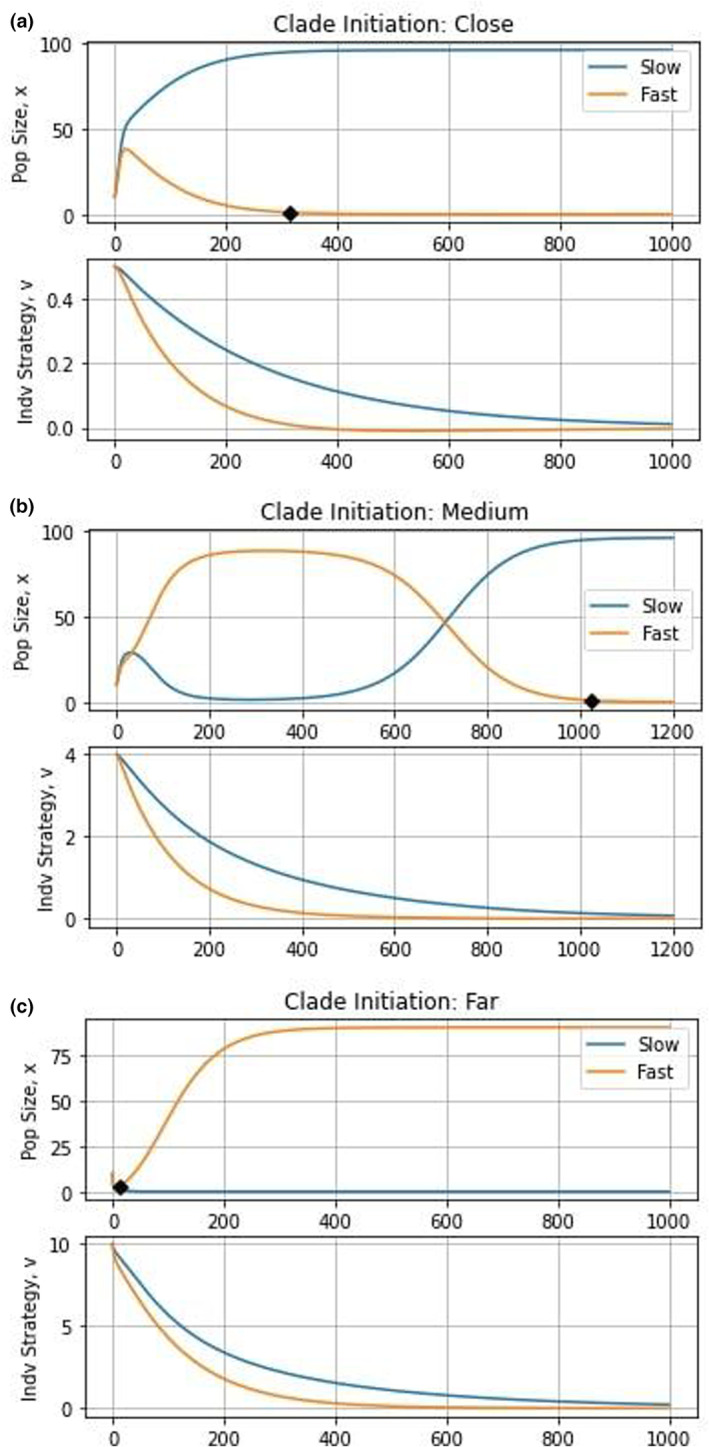
Clade initiation dynamics. The top graphs depict population dynamics and the bottom graphs show strategy dynamics. The orange and blue curves capture the slow‐ and fast‐evolving species, respectively. The black diamond represents the extinction point. The further away from the eco‐evolutionary equilibrium the species start, the more beneficial, for population size, having a higher evolvability is. When species start close to the strategy equilibrium, the fast‐evolving species goes extinct at time unit 315. When they start a medium distance away from the eco‐evolutionary equilibrium, the fast‐evolving species goes extinct at time unit 1022. When they start far from the equilibrium, the slower‐evolving species goes extinct after 11 time units.

Before analyzing the results of the eco‐evolutionary simulations under clade initiation (Figure 1), there are a couple of caveats to note. Since we are using an ODE model, species do not ever truly go extinct but rather reach infinitesimally small population sizes. Thus, we arbitrarily define extinction as occurring when the population density is less than one. In other words, as soon as there is fewer than one individual per unit area (arbitrarily defined), we assume the population goes extinct due to demographic stochasticity or Allee effects. Similarly, coexistence for our purposes generally refers to transient coexistence where the two species maintain sizable populations for prolonged periods of time even as one may eventually outcompete the other. For demonstrative and explanatory purposes, the medium case was chosen to be biased toward preserving the slow‐evolving species.

**FIGURE 2:**
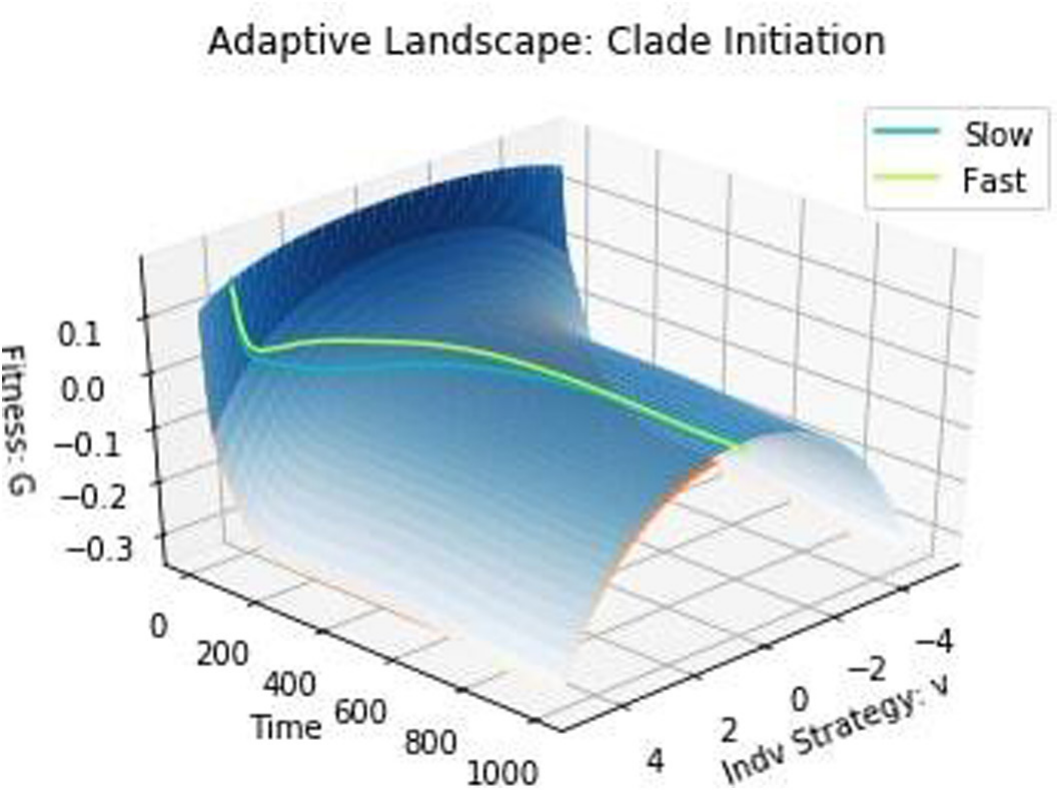
Adaptive landscape: clade initiation. The blue and orange surfaces are the adaptive landscapes for the slow‐ and fast‐evolving species, respectively. The cyan and green‐yellow lines depict the trajectories on the adaptive landscape taken by the slow‐ and fast‐evolving species, respectively. The adaptive landscape is fairly static over time, owing to the quick equilibration of the population and strategy dynamics of both species.

Keeping these adjustments in mind, we see the trends we predicted: as the species' initial strategies move further from the eco‐evolutionary equilibrium, the faster‐evolving species reaches higher densities than the slower evolving one. Therefore, faster‐evolving species do better when far from an eco‐evolutionary equilibrium, whereas slow‐evolving species are more viable when close to an equilibrium. We can visualize these eco‐evolutionary dynamics on each species' adaptive landscape in Figure 2. Because of the cost of evolvability, the adaptive landscape of the slow‐evolving species always lies above that of the fast‐evolving species even as the current strategy of the former species may be higher or lower than the latter species.

As expected, the adaptive landscape is smooth and relatively constant over time, with a single peak for each landscape at v=0. Thus, both $u_{1}$ and $u_{2}$ evolve toward this peak of their respective adaptive landscapes. It is important to note the general trend of the trade‐off between a high and low evolvability rate: a species will benefit from high evolvability that generates much heritable variation when residing on slopes of the adaptive landscape, chasing peaks, and/or occupying new peaks. However, when a species' trait is at a peak, the optimal evolvability value is k=0. If k>0, the species will produce variation in the trait among its population. Since the species already occupies a peak, any variation will, by definition, be off‐peak and result in a lower conferred fitness. To summarize, the closer species are to a peak, the more successful smaller values of k are.

## 4 EVOLUTIONARY TRACKING

**FIGURE 3:**
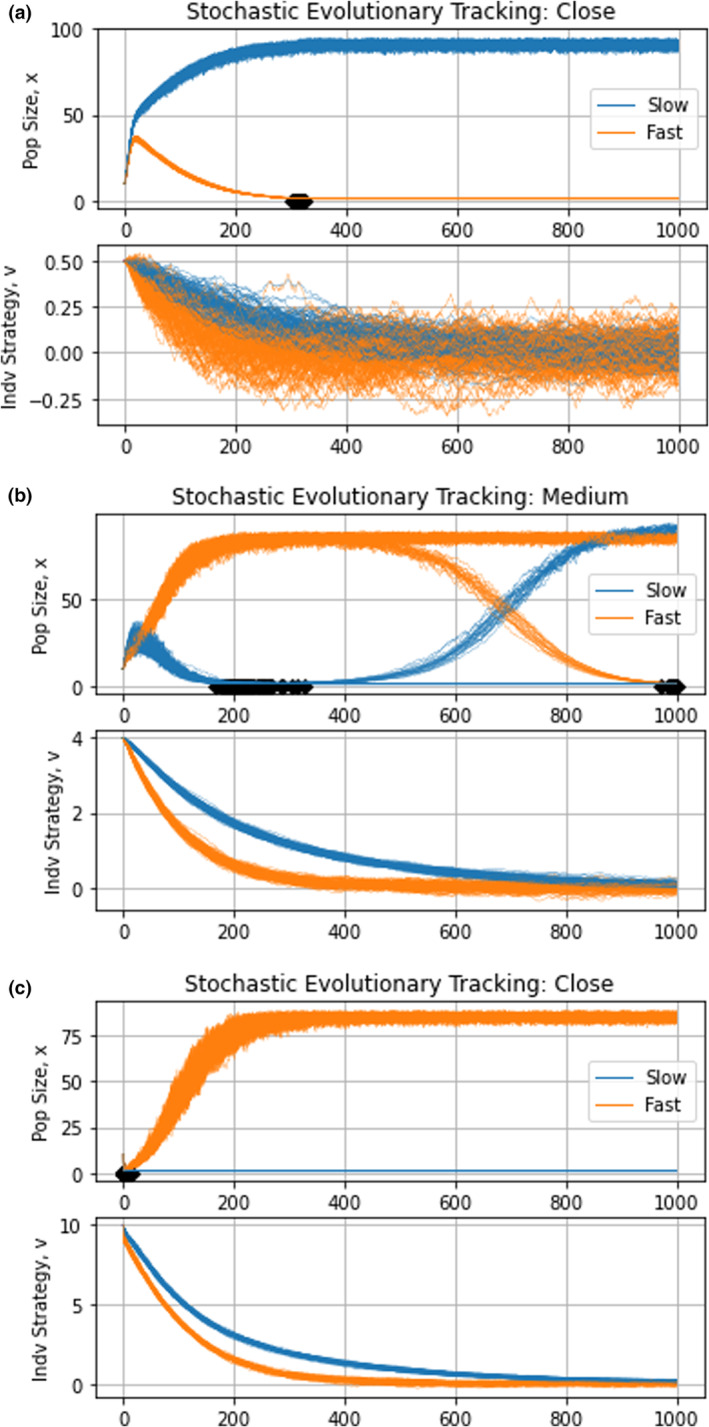
Stochastic evolutionary tracking dynamics. The top graphs depict population dynamics and the bottom graphs show strategy dynamics. Environmental stochasticity speeds up the extinction of species at low population sizes. When species start close to their eco‐evolutionary equilibrium, the fast‐evolving species goes extinct at a mean time of 313.2±4.14 time units. When they start a medium distance away from the equilibrium, the slow‐evolving species went extinct in 77 of the 100 trials at a mean time of 220.1±29.68. When species start far from the equilibrium, the slow‐evolving species goes extinct after 5±3.26 time units.

Environments, from decaying logs to entire ecosystems, are constantly changing on timescales from seconds (e.g., rapid chemical reactions) to millions of years (e.g., changing atmospheric ${\mathrm{CO}}_{2}$ and $O_{2}$ levels since the Cambrian). These changes are sometimes stochastic, like the weather on a day in a tropical rainforest, sometimes predictable like seasons in a year, and sometimes directional over periods of time like ice ages, climate change, and plate tectonics. These changes in a species' environment mean that the peak of the adaptive landscape shifts with time (Vinton & Vasseur, 2020). A trait value that was once on the peak will eventually leave the species on a slope. By the same logic as before, we hypothesize that a species with a higher evolvability will more successfully *track* changes in the peak and its trait value will reside closer to the current optimal trait value (current peak) than one with a lower evolvability. By shifting the trait value which maximizes the carrying capacity, we can model and investigate the advantages of evolvability for evolutionary tracking. This can be done by expanding our equation for carrying capacity:

(8) $$K\left(v\right)=K_{m}\exp\left[-\frac{{\left(v-\gamma \left(t\right)\right)}^{2}}{2\sigma_{k}^{2}}\right]$$

and letting the optimal trait value for maximizing carrying capacity, $\gamma \left(t\right)$, shift with time. Here, we simulate stochastic and deterministic changes to the environment. We simulate the stochastic case in Figure 3 by sampling γ from a uniform distribution between −2 and 2 every 5 time units. Due to the stochastic nature of these simulations, we ran 100 trials for each case. Since we are concerned with evolutionary tracking here, the species will still remain in their fundamental niche (Pienta et al., 2020). Thus, environmental changes were intentionally chosen to be minor but frequent (the effects of drastic but rare environmental changes will be investigated later when we examine evolutionary rescue).

The impact of stochasticity can be seen in the strategy dynamics, with the fast‐evolving species undergoing more abrupt changes in its strategy and tracking the peak more closely than the slow evolving one. However, the results were not what we expected. When species start close or far from the eco‐evolutionary equilibrium, we notice identical qualitative dynamics to the clade initiation case: the fast‐evolving species goes extinct in the former case, whereas the slower‐evolving species goes extinct in the latter. When species start at $u_{1}\left(0\right)=u_{2}\left(0\right)=4$, the fast‐evolving species has an initial advantage over the slow‐evolving species. Unlike the clade initiation scenario, the slow‐evolving species eventually goes extinct in most cases due to stochastic effects. In a minority of cases however, the slow‐evolving species' evolution catches up, its population size recovers, and the fast‐evolving species goes extinct.

Tracking a continually and randomly shifting peak provides little benefit to species in a stochastically changing environment with minor perturbations: since the environmental perturbations are minor and species remain in their fundamental niche. Hence, the fitness cost of being slightly off‐peak is similarly low. The benefits of evolutionary tracking are further buffered because evolving toward a randomly changing fitness peak provides only temporary benefits. The fitness cost of being off‐peak becomes important when either the fast‐evolving species or the slow‐evolving species has a dangerously low population size. When this happens, this cost is enough to push the species to extinction. It is worth noting though, that these results may change with different functional forms for the cost of evolvability and should be viewed with caution awaiting further work in the future.

**FIGURE 4:**
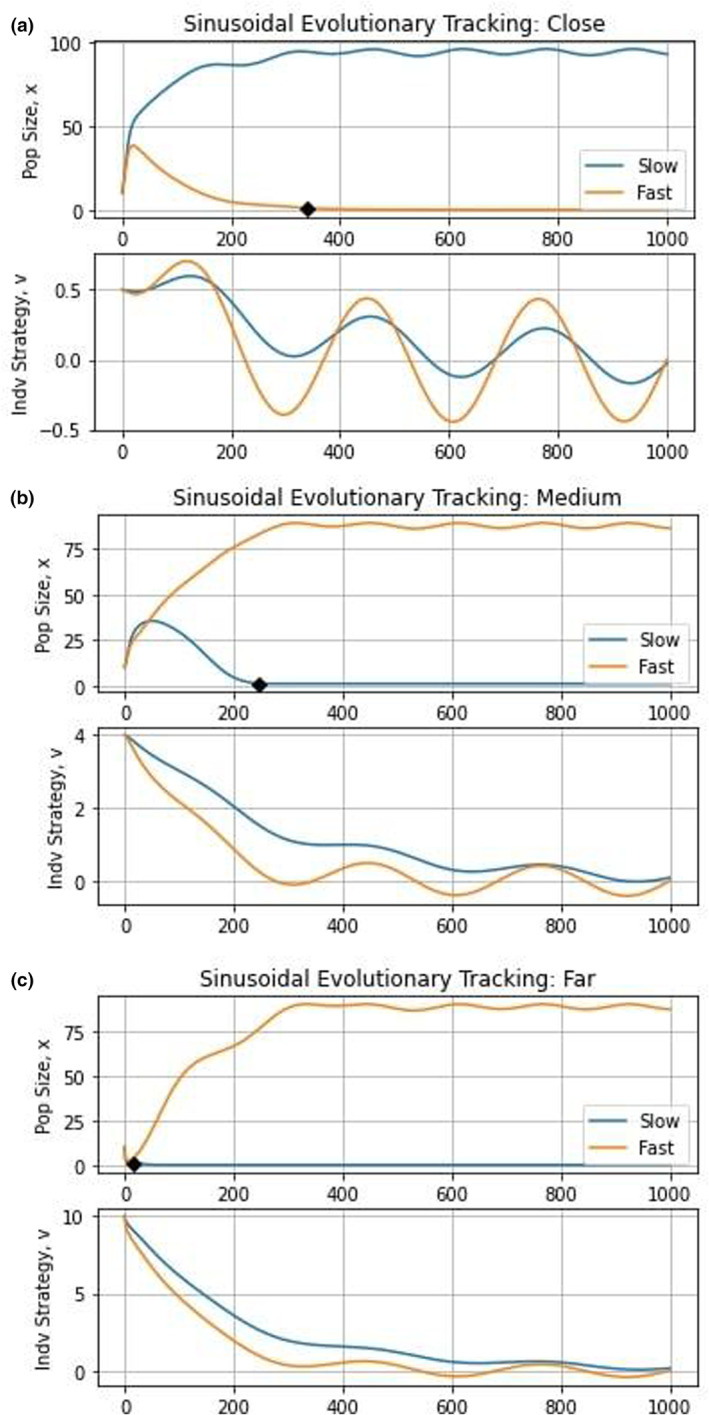
Deterministic evolutionary tracking dynamics. The top graphs depict population dynamics and the bottom graphs show strategy dynamics. Though more tempered than the stochastic case, sinusoidally changing environments promote the extinction of species with low population sizes.

Next, we simulate an environment that changes continually in a periodic fashion, such as changes that occur seasonally or during ice ages, by setting $\gamma \left(t\right)=\sin\left(t/50\right)$. In this case, the slow‐evolving species persists if the species start close to the eco‐evolutionary equilibrium ($u_{1}\left(0\right)=u_{2}\left(0\right)=0.5$) and the fast‐evolving species wins in cases starting medium ($u_{1}\left(0\right)=u_{2}\left(0\right)=4$) and far ($u_{1}\left(0\right)=u_{2}\left(0\right)=10$) from the equilibrium (Figure 4).

Evolutionary tracking under stochastically and periodically changing environments offers striking differences. In the former, with varying degrees of noise, the strategies for both species converge toward u=0. In the latter, the strategies of the two species oscillate between two different extrema with the high evolvability species showing larger oscillations than the low. The frequency and amplitude of variations in the environment and the evolvability of the species determine the strategy dynamics between the fixed values. In comparison to the clade initiation scenario, both forms of evolutionary tracking favor the fast‐evolving species, particularly so for the periodically changing environment. With periodicity, evolutionary tracking is effective and it favors the fast‐evolving species for the two initial conditions of starting at $u\left(0\right)=4$ and $u\left(0\right)=10$. When strategies start near the optimum, $u\left(0\right)=0.5$, the fast‐evolving species persist longer before extinction with periodic environmental variability than either the stochastic environment or clade initiation scenario.

**FIGURE 5:**
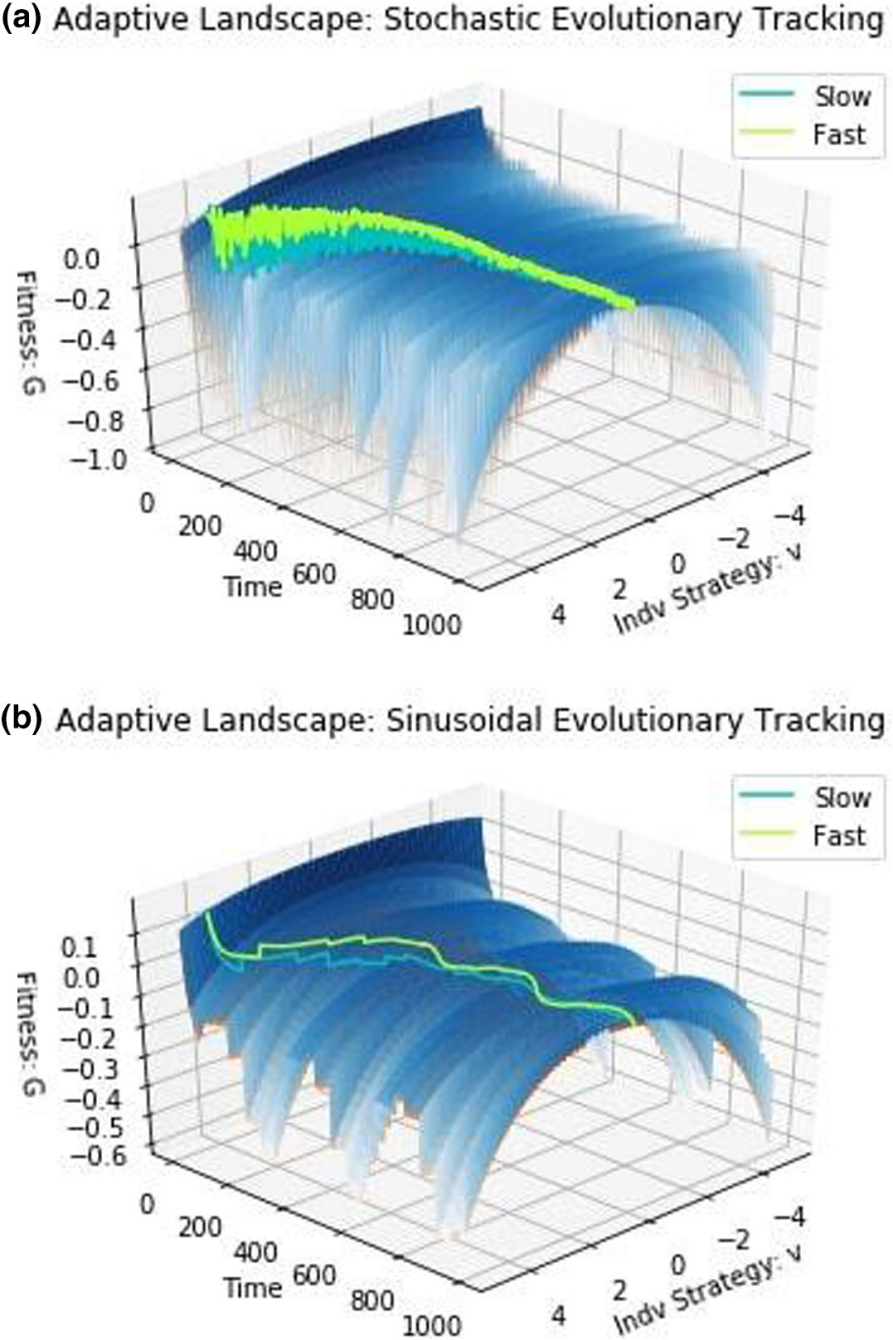
Adaptive landscapes: evolutionary tracking. The highly variable nature of the environment can clearly be seen in the highly irregular shape of the adaptive landscapes. In the stochastic case, there is a convergence in strategy toward the vicinity of u=0, while in the deterministic cases, we see oscillatory behavior in the strategy dynamics.

We consider the adaptive landscapes of the stochastic and deterministic evolutionary tracking cases in Figure 5. As expected, we see a highly irregular landscape with strategies changing constantly. In the stochastic case, there is a convergence toward u close to 0 with noise. This can be clearly contrasted with the deterministically changing environment where the strategies cycle between the values of the upper and lower bounds of the shifting peaks.

## 5 EVOLUTIONARY RESCUE

Catastrophic changes to a species' environment may leave it outside of its fundamental niche where a species' current trait value may not permit its persistence. Namely, ${\left.G\left(v,u_{i},x_{i}\right)\right|}_{v=u_{i}}\ll 0$ for any value of $x_{i}$. For the species to survive, it must evolve fast enough to achieve a viable trait value before its population size drops to some irrecoverably small value of $x_{i}$ (Michor et al., 2005; O'leary et al., 2018; Sansregret & Swanton, 2017). In agriculture, a prime example of this can be seen in the application of chemical or biological agents to deter pests from damaging crops. In this case, pests must achieve some level of pesticide resistance, either physiologically (e.g., rapid excretion of toxins or increased production of enzymes that break down the pesticide; Simon, 2011) or behaviorally (e.g., staying in locations that are not sprayed with pesticide; Gould, 1984), to avoid extinction. All else equal, chances for evolutionary rescue increase with k and x. Thus, we expect that the fast‐evolving species will outcompete the slow‐evolving species since it will be able to evolve an appropriate trait value more before its population goes extinct.

We can model evolutionary rescue by imagining such an abrupt shift in γ that the carrying capacity is now some tiny fraction of $K_{m}$: $K\left(u_{i}\right)<x_{\text{crit}}$ where $x_{\text{crit}}$ is the minimal viable population size for the species. There will be some value of evolvability above which the species is able to evolve a sufficiently changed value of $u_{i}$ such that $K\left(u_{i}\right)>x_{\text{crit}}$ before $x_{i}$ drops to less than $x_{\text{crit}}$. Since we want both populations to be extant when the crisis occurs, we choose to only simulate the case where strategies start a medium distance from equilibrium. Up until the disturbance, this scenario is identical to that of clade initiation starting a medium distance from the equilibrium. We run three simulations, inducing the disturbance by changing γ from 0 to –4 at time unit 600 (when the fast‐evolving species dominates the population), 750 (when the fast‐evolving species and slow‐evolving species have similar population sizes), or 900 (when the slow‐evolving species dominates the population).

**FIGURE 6:**
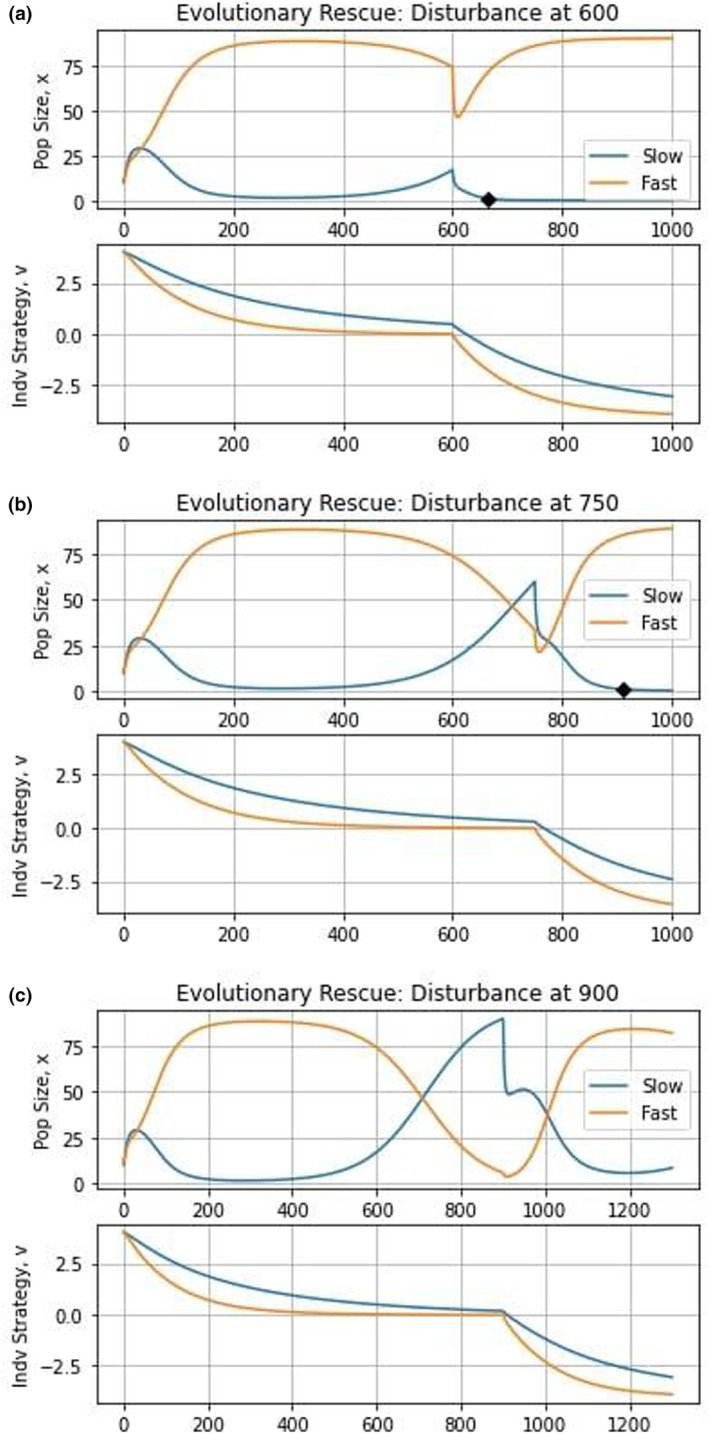
Evolutionary rescue dynamics. The top graphs depict population dynamics and the bottom graphs show strategy dynamics. Regardless of the time the disturbance occurs, the fast‐evolving species outcompeted the slow‐evolving species as it found a viable strategy more quickly.

**FIGURE 7:**
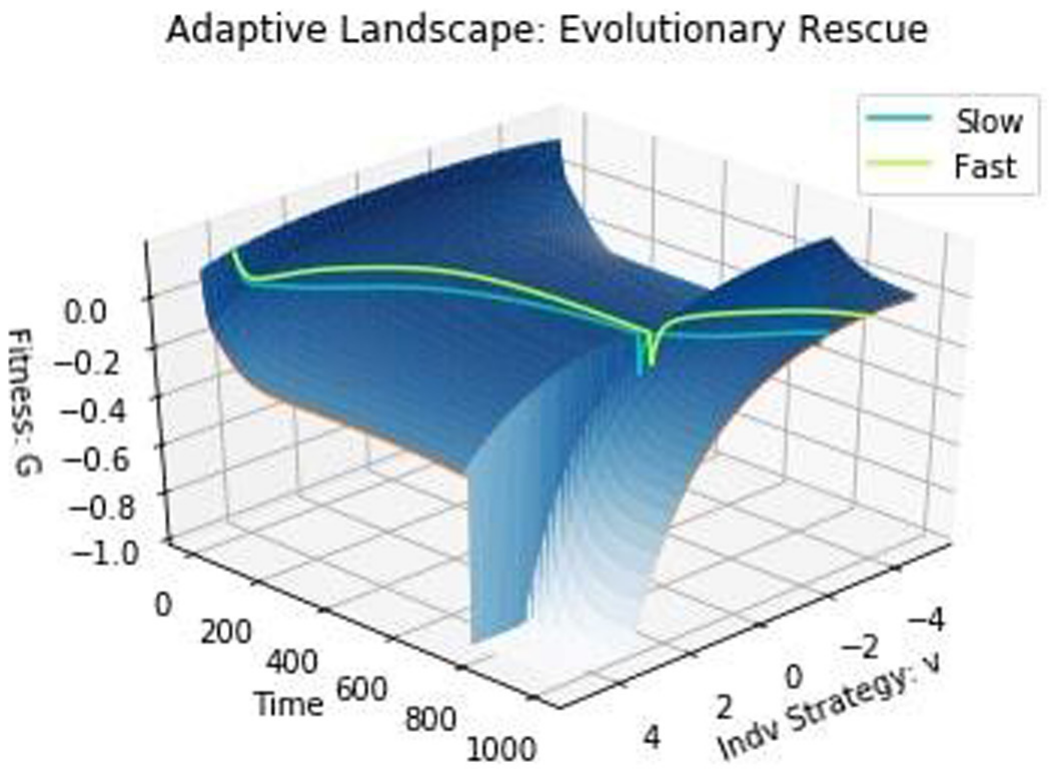
Adaptive landscape: evolutionary rescue. The effect of the disturbance can be seen by the sharp drop in fitness at time 750 and the abrupt change in fitness peak on the adaptive landscape.

From Figure 6, we see that the fast‐evolving species outcompetes the slow‐evolving species in all cases and even drives it to extinction in the first two, at 665 and 910 time units, respectively. Under evolutionary rescue, species are pushed outside their fundamental niche. As a result, the selective pressures to survive environmental stressors are greater than the selective pressures to outcompete other species (Pienta et al., 2020). From the plots of strategy dynamics, we see how the fast‐evolving species was able to respond to the environmental crisis and evolve to a viable equilibrium at –4 more quickly than the slow‐evolving species. In accord with earlier studies (Anciaux et al., 2019; Gomulkiewicz & Holt, 1995; Orr & Unckless, 2014, 2015), this shows that in the face of environmental catastrophe, having a high evolvability is clearly beneficial to stave off extinction. We can see the effects of the environmental catastrophe and of evolutionary rescue on the adaptive landscape shown in Figure 7. Directly after the environment is disturbed, we see a dramatic change in the adaptive landscape, with species rapidly moving from $u_{1},u_{2}\approx 0$ toward *u*_1_,*u*_2_= –4.

When the disturbance occurred at 900 time units (third panel of Figure 6), the slow‐evolving species is not driven extinct, and in time, recovers and eventually outcompetes the fast‐evolving species in the absence of any additional disturbances. This is due to the cost of evolvability, which confers a higher fitness to the slow‐evolving species when they have the same strategy. Transient coexistence becomes possible if each species “stores” fitness gains during good phases to help it survive population loss during bad periods (Adler et al., 2006; Angert et al., 2009; Barabás et al., 2018; Chesson & Warner, 1981; Hallett et al., 2019; Letten et al., 2018; Zepeda & Martorell, 2019).

In the context of our simulations, when periods of stasis are too long, the gains the fast‐evolving species acquired during the times of disturbance are insufficient to allow it to survive. Conversely, when periods of disturbances are too long, the gains of the slow‐evolving species during times of stasis are not enough to allow it to remain extant. However, when “well‐balanced”, both species can effectively utilize their stored gains to allow them to remain in the community. Using this knowledge, we hypothesize that cyclical (transient) coexistence of the species is possible when ecological disturbances occur neither too rarely nor too frequently, but “just right” (Catford et al., 2012; Vandermeer et al., 1996; Wilkinson, 1999).

**FIGURE 8:**
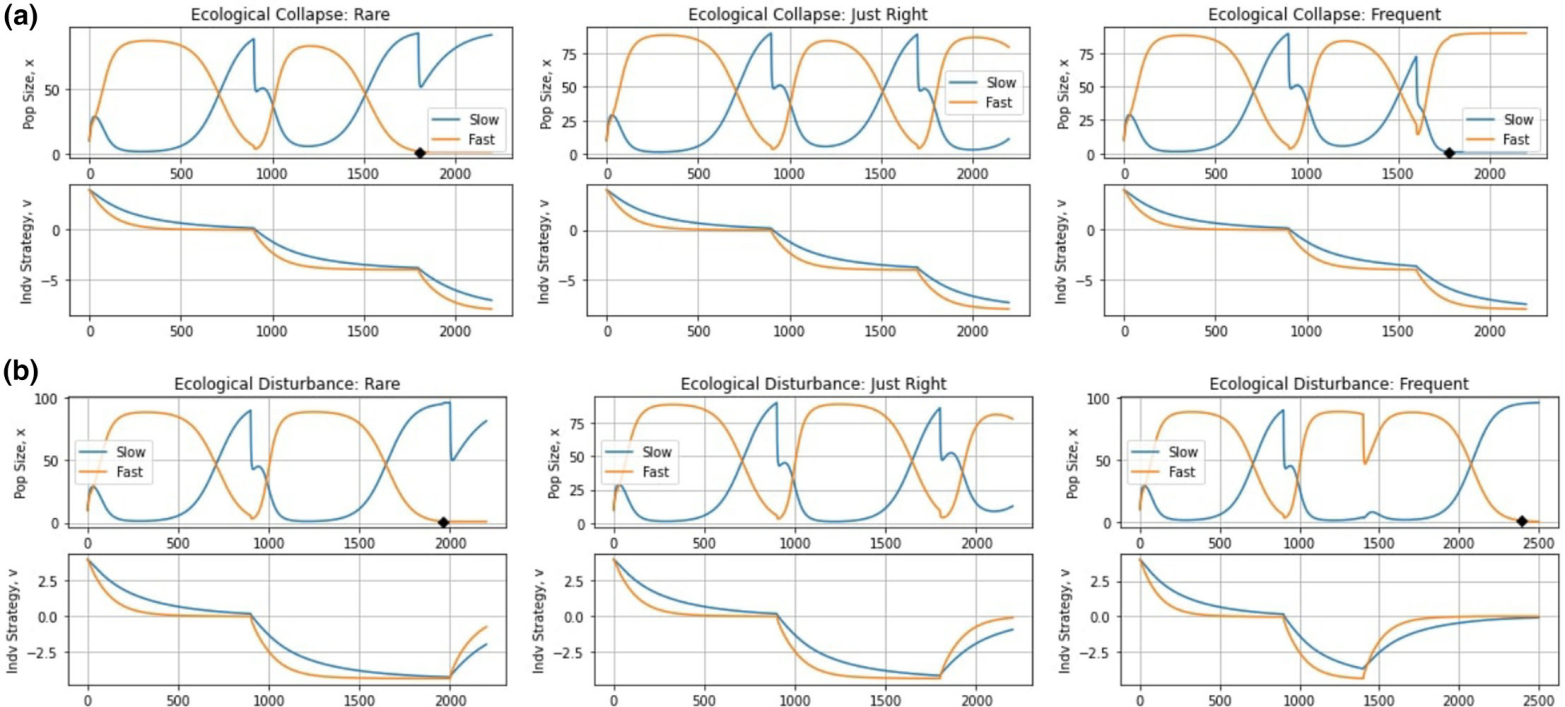
The temporal storage effect: rare, just right, and frequent changes. The top graphs depict population dynamics and the bottom graphs show strategy dynamics. Rarely disturbing the environment promotes its stability, favoring the slow‐evolving species. Frequently disturbing the environment causes instability, favoring the fast‐evolving species. Disturbing the environment at intermediate frequencies favors the fast‐evolving species in periods of disturbance and the slow‐evolving species in periods of stasis, thereby promoting cyclical coexistence.

To further investigate this, we used two sets of simulations, shown in Figure 8. Figure 8a represents ecological collapse from permanent, progressively worsening disturbances from which the population cannot recover to its initial strategy equilibrium, leading to a permanent reduction in carrying capacity for all species. Examples of this include asteroid impacts, abrupt climate change, and large volcanic eruptions. Figure 8b represents more familiar ecological disturbances, such as the application of pesticides, from which populations can recover to their initial equilibria. Three simulations were performed to examine what happens when disturbances to the environment occur rarely, frequently, or at an intermediate frequency.

**FIGURE 9:**
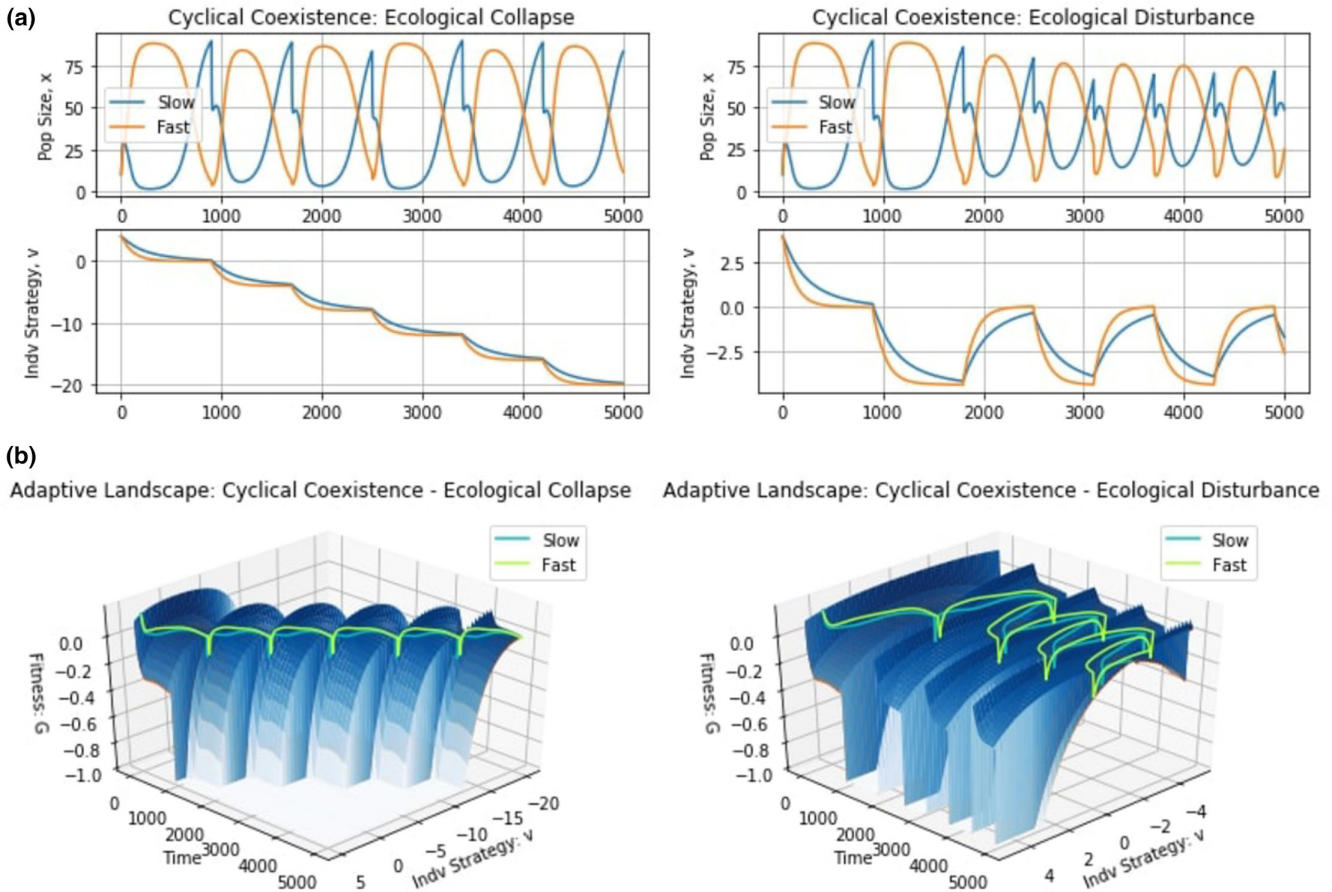
Regular, appropriately timed environmental disturbances. Continually disturbing the environment at intermediate frequencies can lead to the prolonged cyclical coexistence of the fast‐evolving species and the slow‐evolving species.

We can see how timing of catastrophic changes in the environment matters (panels of Figure 9). All three regimes of drastic environmental change had the initial event occur at 900 time units at which point the optimal strategy value shifted from u* = 0 to u* = −4. The regimes then differed in the timing (1800, 1700, and 1600 time units) of a second drastic shift that moved the optimal strategy value to u* = −8. With the lengthier (or shorter) period before the next environmental shift the fast‐evolving species (slow‐evolving species) went extinct. With the intermediate timing of 1700 time units, both species persist together. When disturbances are rare, the environment remains stable long enough to permit both species to spend most of the time near their optimal strategy value. This favors the slow‐evolving species. The opposite happens when disturbances are frequent and species must rapidly change their strategy to persist in which case the slow‐evolving species goes extinct. When disturbances occur at an intermediate frequency, the stable periods favor the slow‐evolving species, while disturbances shift the balance toward the fast‐evolving species. In this way, transient cyclical coexistence of the two species can occur. Note this coexistence is not for two species with fixed traits but rather requires the eco‐evolutionary dynamics that comes with large and abrupt shifts in the peak of the adaptive landscape. Furthermore, note that disturbance timings were chosen manually at intermediate frequencies to promote coexistence.

Modeling a transient ecological disturbance yields similar results to the successive catastrophes (Figure 8b). The initial disturbance at time 900 shifts the optimal strategy to u*= –4.4. When the prolonged disturbances cease at 2000, 1800, and 1400 time units and return the optimal strategy to $u^{*}=0$, then the fast‐evolving species goes extinct, the two species transiently coexist, and the fast‐evolving species goes extinct, respectively. As before, rare disturbances favor the slow‐evolving species and vice versa for more frequent disturbances. Transient coexistence becomes possible at the intermediate value as each species suffers in different ways from the on–off cycle of disturbances. Namely, the switching between the disturbed state (perhaps following a volcanic eruption, or with the onset of an ice age) and the undisturbed state (following the dissipation of the disturbed state) creates an opportunity for transient coexistence simply based on the eco‐evolutionary dynamics and there is a cost of evolvability. Transient coexistence based on the evolutionary dynamics and frequency of ecological collapse or disturbances occurs on the shifting adaptive landscapes as each species finds itself requiring evolutionary rescue with each shift in peak, and then rechallenged even as each species begins to approach the new peak (Figure 9). Again, note that disturbance timings were chosen manually at intermediate frequencies to promote coexistence.

When environmental disturbances occur at intermediate frequencies, transient cyclical coexistence of the fast‐evolving species and slow‐evolving species is possible. This mirrors early work showing how nonequilibrium population dynamics can promote coexistence of two species where one is superior at low‐population densities and the other at high‐populaiton densities. In our case, the trade‐off between evolvability and competitive ability promotes coexistence. In this way, our result mirrors that of Yamamichi and Letten (Yamamichi & Letten, 2021). Their consumer‐resource model showed how a fast‐evolving species (adjusting foraging parameters to resource level) can coexist with one that does not evolve. Coexistence requires the evolver to have a higher subsistence resource level, $R^{*}$, than the nonevolver. By way of difference, in the Yamamichi and Letten model (Yamamichi & Letten, 2021), fluctuations emerge from intrinsic nonequilibrium resource dynamics, whereas in our model, we imagined an external driver of cyclic population dynamics.

## 6 ADAPTIVE RADIATION

Adaptive radiations describe the rapid diversification of organisms from an ancestral species into several species. This occurs when the environment changes to open new resources or opportunities for niche partitioning, when a novel taxon invades a new region, or when a taxon experiences a constraint‐breaking adaptation (Schluter, 2001; Stroud & Losos, 2016; Yoder et al., 2010). An adaptive radiation occurs by modifying an important trait through which the species interact with their environment such as beak size (e.g., Galapagos finches and Hawaiian honeycreepers) or head shape (e.g., cichlids of the African great lakes). As species diverge in their trait values, interspecific competition declines (Ronco et al., 2021; Takahashi & Koblmüller, 2011; Tebbich et al., 2010). In the case of Darwin's finches, all species arose from a single ancestral species that arrived in the Galapagos some 3 million years ago and rapidly diversified into the approximately 15 species observed today (De León et al., 2014; Foster et al., 2008). In the context of eco‐evolutionary dynamics, this implies that there are currently multiple valleys and peaks on the adaptive landscape of Darwin's finches.

Within the framework of G functions or adaptive dynamics, the cascade of speciation events can involve a species and subsequent species evolving to convergent stable minima of the adaptive landscape (also known in game theory as evolutionary branching points; Geritz et al., 1998). When the trait value of a species evolves to such a minimum disruptive selection may promote two daughter species through adaptive speciation (Cohen et al., 1999; Doebeli & Dieckmann, 2000). Four features have been theorized to identify adaptive radiation (Schluter, 2001). (1) A common, recent ancestry for the species. We model this as the species sharing the same G‐function and starting with a single species with a particular strategy value. (2) A phenotype–environment correlation. We model this by letting the strategy *u* represent a trait influencing the exploitation of an environmental niche axis. (3) The trait impacts the fitness of the species. This requires that the individual's strategy value, *v*, influences its fitness. (4) Speciation. This can occur through adaptive speciation at a convergent stable minimum. By incorporating these four features into each species G function, we expect the fast‐evolving species to speciate and fill niches faster and ultimately become more diverse than the slow‐evolving species.

The breadth of the competition function, $\sigma_{a}^{2}$, influences the number of peaks at the ESS: Lower values of $\sigma_{a}^{2}$ promote a greater number of species at the ESS. By adjusting $\sigma_{a}^{2}$, we can use our model to simulate adaptive radiation and evaluate the role of evolvability in determining the rate at which the fast‐ and slow‐evolving species diversify. To avoid artifacts such as arbitrary coexistence and infinite niche packing that result from using Gaussian functional forms for competition and carrying capacity, and when $\sigma_{a}^{2}<\sigma_{k}^{2}$, we let $a=0.05+0.95\left(\exp\left[-{\left(v-u_{i}\right)}^{2}/2\sigma_{a}^{2}\right]\right)$ (Barabás et al., 2012, 2013; Cressman et al., 2017; Gyllenberg & Meszena, 2005).

**FIGURE 10:**
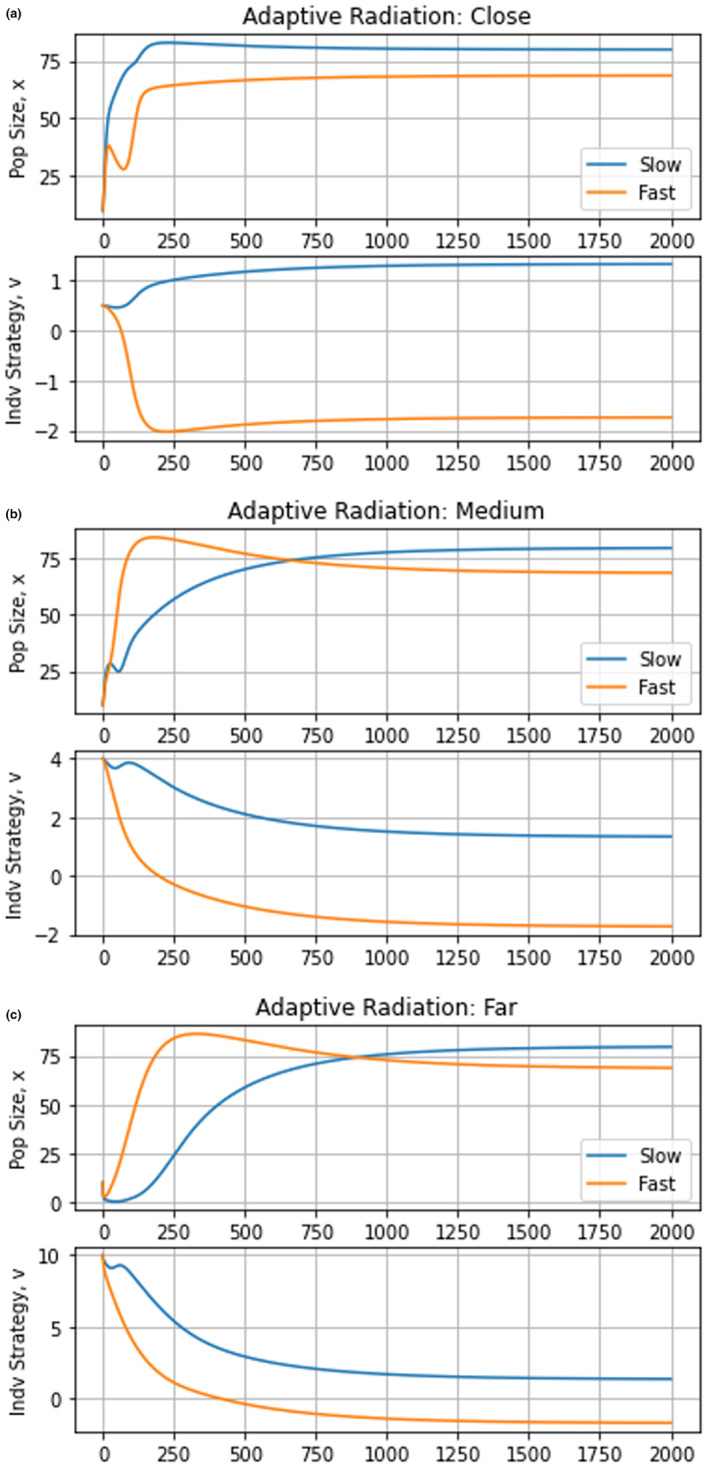
Adaptive radiation dynamics. The top graphs depict population dynamics and the bottom graphs show strategy dynamics. To avoid competition, species occupy distinct niches. The slower‐evolving species reaches slightly higher densities than the faster‐evolving one in all cases.

**FIGURE 11:**
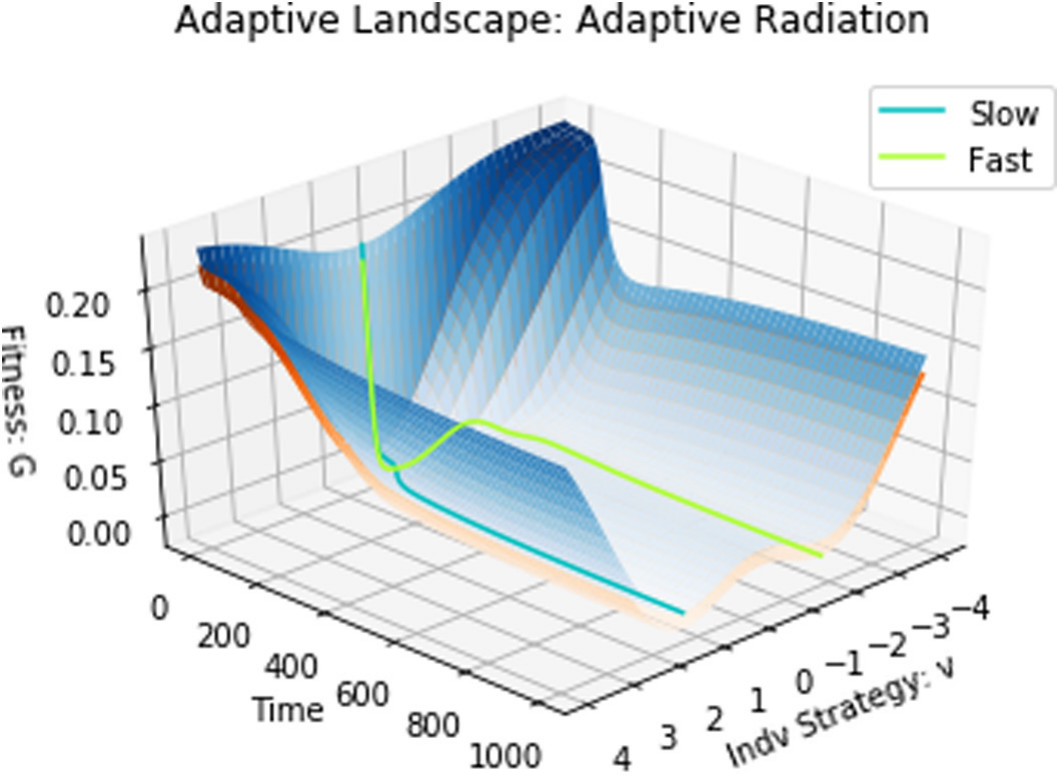
Adaptive landscape: adaptive radiation. There is a clear divergence in strategy values, with species evolving toward distinct convergent stable minima on the adaptive landscape.

**FIGURE 12:**
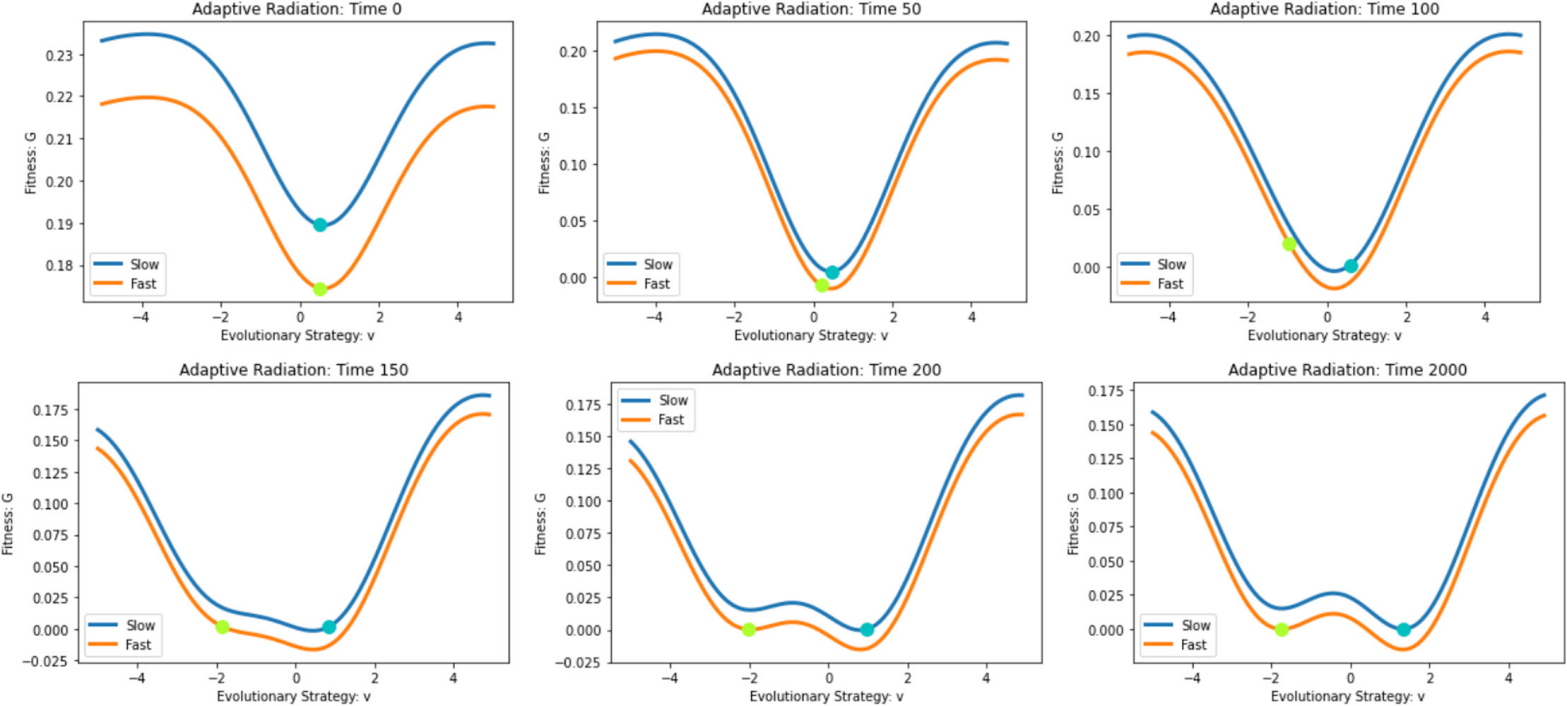
Two‐dimensional adaptive landscapes: adaptive radiation. The fast‐evolving species and slow‐evolving species (depicted by the green–yellow and cyan dots, respectively) start at a valley of their adaptive landscapes and begin scaling their adaptive landscapes in opposite directions. Eventually, two local minima emerge in each adaptive landscape. At the end of the simulation, the species converge to two different minima with the potential for evolutionary branching and further speciation.

By setting $\sigma_{a}^{2}=2$, the model shifts from a single peak of the adaptive landscape at the ESS to a model with infinite niche packing. If we start with a single fast‐ and slow‐evolving species, the eventual eco‐evolutionary equilibrium remains the same regardless of whether we start both species at $u\left(0\right)=0.5$, $u\left(0\right)=4$, or $u\left(0\right)=10$ (Figure 10). The equilibrium has both species coexisting with strategy values at different convergent stable minima (Figures 11 and 12). This result satisfies the first three conditions for adaptive radiation.

Because of the cost of evolvability, the adaptive landscape of the slow‐evolving species lies above that of the fast evolver (Figure 12). If a second species belonging to the G function of the slow evolver were to be introduced at the $u^{*}$ of the fast evolver, it would outcompete the fast evolver and the community would be left with two slow‐evolving species coexisting at distinct convergent stable minima. But, such an invasion may not occur. Instead, upon converging on a stable minimum the species, be it a fast or slow evolver, suggests the possibility for evolutionary branching and further speciation (Champagnat & Méléard, 2010; Dieckmann & Ferrière, 2009; Doebeli & Dieckmann, 2000; Geritz et al., 1998; Wakano & Iwasa, 2013).

To simulate the adaptive radiation, we implemented a manual procedure for adding species to the community. First, we started with two species, one each from the fast‐ and slow‐evolving G functions. Next, we let them evolve toward their respective convergent stable minima. As soon as a species' strategy was within 0.02 of its minimum, it was allowed to speciate with the daughter species having a strategy value ±0.02 of the parent species' strategy (randomly drawn from a uniform distribution), and an initial population size of 1. Once the new species was introduced, the simulation was continued with this additional species until a species again approached within 0.02 of a convergent stable minima at which point the process of speciation was repeated.

**FIGURE 13:**
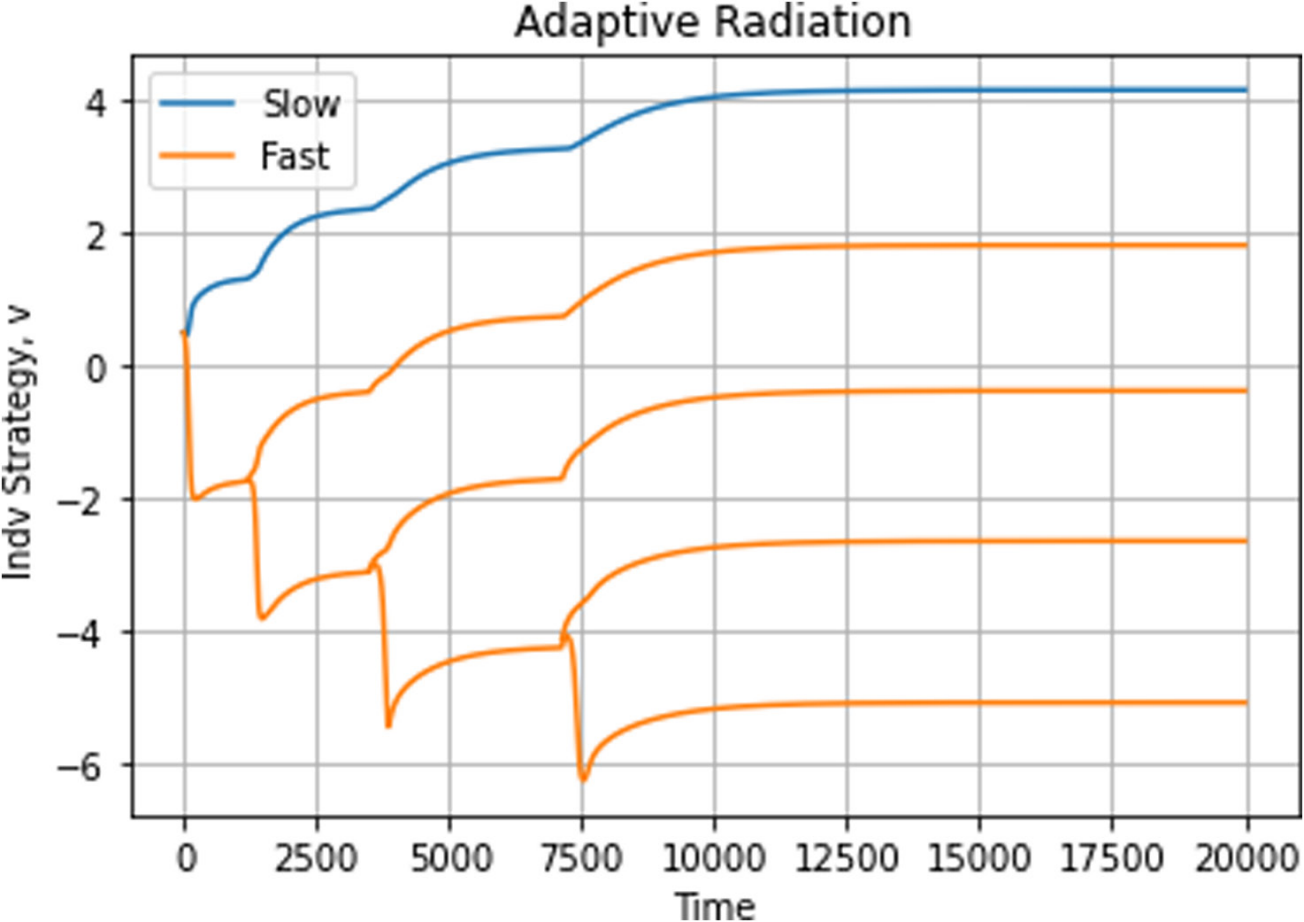
Adaptive radiation phylogeny. When slow‐ and fast‐evolving species are introduced into an environment with a potential for adaptive radiation (low $\sigma_{a}^{2}$), the fast‐evolving species undergoes rapid radiation, whereas the slow‐evolving species is left scaling its peak, which is continually perturbed by the speciation of the faster‐evolving species.

Despite the cost of evolvability, the fast‐evolving species undergoes the radiation and the slow evolver does not (Figure 13). Initially, the fast‐evolving species converges on its minima faster than the slow evolver. Thus, it speciates first, and in so doing disrupts the adaptive landscapes with the addition of another species. Once again there is an evolutionary race toward the convergent stable minima. Each time, a fast‐evolving species gets to its minima before the slow‐evolving species. With time there are more and more fast‐evolving species that spread further across the strategy space. The fast evolver that determines the next speciation event seems somewhat haphazard with respect to strategy value. But, two trends hold true. First, with each speciation event, we see diffuse coevolution as each species' strategy diverges from its nearest competitor species. Second, the slow evolver never goes extinct. It simply evolves a more extreme strategy value with each speciation event, and it is never the first to evolve to a new minimum. As expected, evolvability can be favored when evolutionary branching results in adaptive radiation.

## 7 SUMMARY

We summarize the main findings for each of the four evolutionary scenarios presented above:

*Clade initiation*: The further away species are from an eco‐evolutionary equilibrium, the more beneficial it is to have a high evolvability.

*Evolutionary tracking*: Frequent minor environmental changes promote the extinction of species with small population sizes.

*Evolutionary rescue*: Faster‐evolving species respond better to drastic changes in the environment, whereas slower‐evolving ones fare better in static environments. Disturbing the environment at appropriate frequencies and levels allows for transient cyclical coexistence of fast‐ and slow‐evolving clones.

*Adaptive radiation*: When there are multiple niches of equal quality in an environment for species to occupy, species can coexist by having divergent trait values. Furthermore, the costs of evolvability likely outweigh the benefits; consequently, slower‐evolving species often perform slightly better.

## 8 CONCLUSION

In this paper, we created a model of two competing populations: the fast‐evolving species and slow‐evolving species and endowed them with high and low evolvabilities, respectively. We subjected these species to different selective pressures and analyzed population and strategy dynamics. The G function framework that we use is closely related to other quantitative genetics approaches. Apart from the focus on competing species rather than predator–prey dynamics, it is identical in formalism to (Mougi & Iwasa, 2011) and is closely related to the approaches outlined in (Raatz et al., 2019; van Velzen & Gaedke, 2018), except for the lack of negative exponential boundary functions to prevent negative strategy values (an issue we ignore due to the theoretical and abstract nature of our model). In each of these approaches, the speed of evolution changes dynamically as the selection gradient changes, but evolvability is kept constant. A critical difference between the G function approach to eco‐evolutionary dynamics and the quantitative genetics approaches mentioned above is the difference between genetic evolution and plasticity. Under the G function framework, we assume the evolutionary dynamics strictly refer to genetic evolution, whereas plasticity is incorporated by changing the model formalism into that of a structured population (Bukkuri, 2023; Bukkuri & Brown, 2023; Bukkuri et al., 2022a, 2022b; Cunningham et al., 2021). However, the other quantitative genetic approaches (Raatz et al., 2019) make no such distinction.

From our clade initiation simulations, we found that the further away the species started from their strategy equilibria, the better the fast‐evolving species (the species with high evolvability) fared. Continual, minor environmental changes (both stochastic and periodic) promoted the extinction of species with low population sizes irrespective of evolvability. In these simulations, rapid evolutionary tracking, made possible by high evolvability, was found to confer a negligible benefit. We saw that the fast‐evolving species responded much better to abrupt changes in the environment, undergoing evolutionary rescue more effectively than the slow‐evolving species. Taking into account these findings, we demonstrated a method under which transient coexistence of the slow‐evolving species and fast‐evolving species was possible. During adaptive radiations when several niches were available for the species to occupy, we found that fast‐evolving species were able to radiate rapidly. This left the slow‐evolving species chasing its peak, continually perturbed by speciation events of the faster‐evolving species.

## AUTHOR CONTRIBUTIONS

**Anuraag Bukkuri:** Conceptualization (lead); formal analysis (lead); funding acquisition (equal); investigation (lead); methodology (lead); software (lead); writing – original draft (lead); writing – review and editing (equal). **Kenneth J. Pienta:** Conceptualization (equal); formal analysis (equal); funding acquisition (equal); project administration (equal); supervision (equal); writing – review and editing (equal). **Sarah R. Amend:** Conceptualization (equal); funding acquisition (equal); project administration (equal); supervision (equal); writing – review and editing (equal). **Robert H. Austin:** Conceptualization (equal); funding acquisition (equal); project administration (equal); supervision (equal); writing – review and editing (equal). **Emma U. Hammarlund:** Conceptualization (equal); funding acquisition (equal); project administration (equal); supervision (equal); writing – review and editing (equal). **Joel S. Brown:** Conceptualization (equal); formal analysis (supporting); funding acquisition (equal); methodology (supporting); project administration (equal); resources (equal); validation (supporting); writing – review and editing (equal).

## CONFLICT OF INTEREST STATEMENT

KJP is a consultant for CUE Biopharma, Inc. and holds equity interest in CUE Biopharma, Inc., Keystone Biopharma, Inc. and PEEL Therapeutics, Inc. SRA holds equity interest in Keystone Biopharma, Inc. AB, RHA, EUH, and JSB declare no potential conflict of interest.

## Data Availability

Codes used to produce plots in this paper can be found at https://github.com/abukkuri/Evolvability.
